# Real-time arrhythmia detection using convolutional neural networks

**DOI:** 10.3389/fdata.2023.1270756

**Published:** 2023-11-20

**Authors:** Thong Vu, Tyler Petty, Kemal Yakut, Muhammad Usman, Wei Xue, Francis M. Haas, Robert A. Hirsh, Xinghui Zhao

**Affiliations:** ^1^School of Engineering and Computer Science, Washington State University, Vancouver, WA, United States; ^2^Department of Mechanical Engineering, Rowan University, Glassboro, NJ, United States; ^3^Department of Anesthesiology, Cooper University Hospital, Camden, NJ, United States

**Keywords:** big data, smart health, machine learning, anomaly detection, convolutional neural networks

## Abstract

Cardiovascular diseases, such as heart attack and congestive heart failure, are the leading cause of death both in the United States and worldwide. The current medical practice for diagnosing cardiovascular diseases is not suitable for long-term, out-of-hospital use. A key to long-term monitoring is the ability to detect abnormal cardiac rhythms, i.e., arrhythmia, in real-time. Most existing studies only focus on the accuracy of arrhythmia classification, instead of runtime performance of the workflow. In this paper, we present our work on supporting real-time arrhythmic detection using convolutional neural networks, which take images of electrocardiogram (ECG) segments as input, and classify the arrhythmia conditions. To support real-time processing, we have carried out extensive experiments and evaluated the computational cost of each step of the classification workflow. Our results show that it is feasible to achieve real-time arrhythmic detection using convolutional neural networks. To further demonstrate the generalizability of this approach, we used the trained model with processed data collected by a customized wearable sensor from a lab setting, and the results shown that our approach is highly accurate and efficient. This research provides the potentials to enable in-home real-time heart monitoring based on 2D image data, which opens up opportunities for integrating both machine learning and traditional diagnostic approaches.

## 1 Introduction

Heart disease is a key health problem worldwide; in the United States alone, over 850,000 people die each year from cardiovascular diseases (Virani et al., 2020). Associated costs run into the hundreds of billions of dollars. Between 2014 and 2015, the US economy spent almost $219 billion on diagnosis and treatment of heart disease (Fryar et al., 2012), and in particular, direct medical costs associated with congestive heart failure (CHF) amounted to $20.9 billion in 2012 (Cook et al., 2014). These are expected to increase to $53 billion by 2030 (Heidenreich Paul et al., 2013), with the majority of costs related to hospitalization. However, hospitalizations may be avertible provided patients and clinicians are cued to intervene prior to significant deterioration in cardiac functions. By acting upon an “early warning,” a clinician could remotely instruct a patient to adjust a intake of fluids, salt, or medication, thereby avoiding an *avoidable* hospitalization or informing just-in-time palliative care strategies (Gadoud et al., 2013). This is becoming ever more realizable with the recent advances in the Internet of Things (IoT) and data analytics. Healthcare is increasingly leveraging information technologies for more efficient diagnostics and treatment by “smart” and “connected” systems (Akmandor and Jha, 2018; Greco et al., 2020). These systems provide intelligent services for health monitoring and medical automation in different contexts and environments, e.g., home, on-the-go, etc., permitting substantial reduction in cost of care (Ren et al., 2015; Kumar and Rajasekaran, 2016).

Many heart conditions exhibit biophysical signals that can be detected before acute, irreversible damage is sustained by the heart or before more extensive damage is incurred, thereby reducing adverse health events. For example, various types of arrhythmia, can be monitored using home-based/mobile health (m-Health) monitoring platforms based on single-mode sensing (i.e., electrocardiogram (ECG), Singh and Jasuja, 2017; Ahsanuzzaman et al., 2020; Xu, 2020). In our prior work (Yakut et al., 2022), we designed a rechargeable, compact, and wearable heart health monitor that acquires real-time ECG from the human body. Recorded information can be transferred wirelessly to the user's phone or computer, where a machine learning model can be used to monitor biophysical data and identify anomalies. The relatively inexpensive cost of this device enables home monitoring in many cases, if real-time ECG data processing can be implemented.

Existing studies on applying big data and machine learning technologies on ECG data for arrhythmic detection often focus on the learning performance, such as accuracy, precision, etc., instead of the potentials of supporting real-time processing. Some early work on real-time analysis of ECG data uses 1-D representation coupled with time-series based processing to achieve higher computational performance (Petty et al., 2020; Zhou et al., 2020; Bertsimas et al., 2021). However, representing ECG data in 1D loses the rich 2D features, and may hinder the potential of integrating data analytics technologies with traditional diagnostic approaches. In this paper, we present a different approach in supporting real-time arrhythmic detection using convolutional neural networks, aiming to preserve the 2D features and also satisfy the real-time processing needs. Specifically, we have developed a small-scaled convolutional neural network and trained the model using images of short duration ECG samples. We have carried out extensive experiments to evaluate the capability of a neural network running in real-time. This approach has an average accuracy of 90% and an average classification time of about 0.02 s (20 ms). The proposed method can be applied to a wearable sensor, where the sensors collect raw data from an individual and the data is then passed onto the neural network to analyze data in real-time. The proposed method is a small-scaled convolutional neural network that is feasible to execute on resource-constraint devices to support in-home monitoring systems. This allows for faster diagnostic of arrhythmia and can be deployed in a medical environment without powerful hardware.

The rest of the paper is organized as follows. Section 2 reviews related work. Section 3 describes the dataset that was used to train and test the proposed approach. Section 4 presents the convolutional neural network using image-based inputs, and its evaluation. Section 5 explains the workflow for ECG detection to evaluate its capability of meeting real-time processing needs. Section 6 shows results using real world data collected from our sensing platform. Finally, Section 7 concludes the paper and presents future directions for this research.

## 2 Related work

Cardiovascular diseases are the leading cause of death both in the U.S. and worldwide. The direct domestic medical costs associated with congestive heart failure (CHF) are expected to reach $53 billion by 2030 (Heidenreich Paul et al., 2013), with the majority of costs related to hospitalization. However, hospitalization may be avertible provided patients and clinicians are cued to intervene prior to significant deterioration in cardiac functions. Long-term and realiable in-home monitoring is needed to address these challenges. Cardiac monitoring using ECG electrodes and bedside monitors has been implemented in the medical field for over 70 years. The standard 12-lead ECG, along with other reduced-lead (5- or 3-electrode) configurations, can accurately measure signals and help diagnose complex heart conditions (Drew et al., 1999; Petrenas et al., 2015; Francis, 2016; Zègre-Hemsey et al., 2016). Various machine learning approaches have been applied for predicting cardiovascular diseases (Krittanawong et al., 2017; Altan et al., 2018a,b, 2021; Shameer et al., 2018; Vocaturo and Zumpano, 2021). One of the most well-known and popular methods used to classify ECG data is a Support Vector Machine (SVM) (Zadeh et al., 2010; Li et al., 2015; Dinakarrao et al., 2019) with various kernels, feature extraction methods, and categories of arrhythmia.

For neural network approaches, a common method is to use a convolutional neural network. Güler and Übeyl propose a method for data engineering for ECG signals, by using discrete wavelet transform (DWT) to extract additional information about the signal in the form of wavelets (Güler and Übeyli, 2005). These wavelets, in addition to a few statistical features derived from the signal, are used in a modular neural network, where each input of the network has its own neural network and work independently of each other. This network works because DWT can be broken down multiple times and each wavelet can be learned by the network. A 34-layer deep residual neural network is proposed in Rajpurkar et al. (2017). Beside the 1D-convolutional approaches, 2D-convolutional approaches exist (Acharya et al., 2018; Wu et al., 2018; Dinakarrao et al., 2019). For example, Jun et al. (2018) extracts an image from an ECG sample and uses a 2D-convolutional neural network to learn patterns from images. The network architecture of their proposed work is similar to that found in existing deep learning image models such as VGGNet (Simonyan and Zisserman, 2014).

Real-time decision making is the key requirement of many emerging applications that pose a set of new challenges to the deployment of machine learning models (Nishihara et al., 2017). Specifically, machine learning models must be able to operate with low latency (Crankshaw et al., 2015) and high throughput (Nair et al., 2015; Silver et al., 2016), in order to satisfy the real-time requirement of these applications. In the area of cardiovascular medicine, initial efforts have been made toward real-time processing of ECG signals to diagnose relevant diseases (Jin et al., 2009; Oresko et al., 2010). To this end, processing and analyzing ECG data as time series attracts increasing attention, and long short-term memory networks (Petty et al., 2020) have been used to achieve higher performance.

The existing studies on real-time processing have not used CNN based 2D approaches, due to its deep architecture which presents challenges for meeting the real-time processing needs. However, preserving 2D features offers the potential of integrating machine learning based approaches with the traditional diagnostic approaches, which opens up a broad range of opportunities to enhance the at-home monitoring. In this paper, we bridge this gap by exploring the potentials of using CNN based approaches to support real-time arrhythmic detection. Specifically, we developed and evaluated a simple 2D CNN that is trained by ECG images. The baseline architecture for this model is derived from existing work, with the model size reduced to address the challenge of a resource-constrained environment. We then develop a simulator to mimic the on-going monitoring, where the data are generated continuously and the detection must be done on the fly. This approach is evaluated using both open-source data and data collected in a lab setting using our wearable sensor prototype (Yakut et al., 2022).

## 3 Dataset

In this paper, we used the MIT-BIH Arrhythmia Database (Goldberger et al., 2000) to train and evaluate our machine learning models. The database provides 48 records of different individuals, with varying age and medical conditions. Each record includes a 30-minute ECG recording recorded in two channels at a rate of 360 Hz (samples per second). The database provides a list of annotations that describes what conditions have been diagnosed in the nearby ECG region, as well as where the conditions are located. The full list of annotations contains various annotations that are labeled as single characters. These labels are divided into two categories, beat and non-beat annotations. Beat annotations describe the heartbeat and non-beat may describe the start/end to a region, peaks, and comments.

The original MIT-BIH Arrhythmia Database contains 40 different annotations for heartbeats. However, many of the annotations are not useful in classifying arrhythmic conditions. To improve the learning process, we grouped the non-beats annotations into one annotation (annotation “Z”). Table 1 shows the description of the annotations that are found in the database.

**Table 1:** List of annotations for classification.

**Annotation**	**Description**
N	Normal beat
L	Left bundle branch block beat
R	Right bundle branch block beat
V	Premature ventricular contraction
A	Atrial premature contraction
f	Fusion of paced and normal beat
F	Fusion of ventricular and normal beat
j	Nodal (junctional) escape beat
a	Aberrated atrial premature beat
E	Ventricular escape beat
J	Nodal (junctional) premature beat
Q	Unclassifiable beat
e	Atrial escape beat
S	Premature or ectopic supraventricular beat
Z	Non-Beat

**Figure 1:**
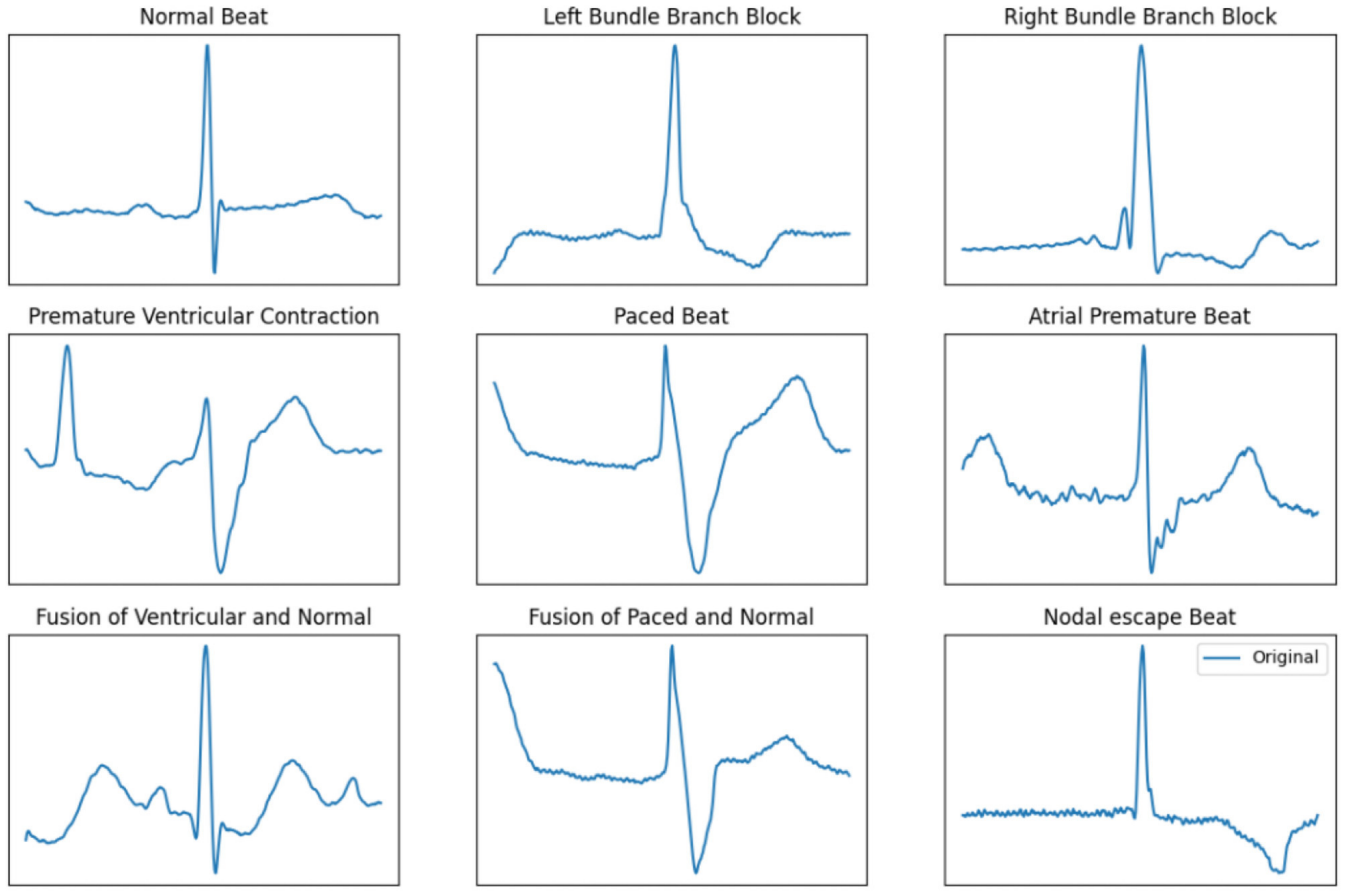
ECG signals of nine different types of heartbeat.

Figure 1 shows the ECG signals of nine different types of heartbeats, with the first being a normal beat while the remaining are arrhythmia. As the figure shows, the overall pattern of any arrhythmia differs from the normal beat, making it possible for a classification algorithm to recognize and classify different ECG patterns.

## 4 Arrhythmic detection using convolutional neural networks

### 4.1 Data preprocessing

Preserving the 2D features of ECG signals provides the potential of integrating machine learning detection with the traditional diagnostic approaches. Therefore, the first step of our work is to preprocess the data from the MIT-BIH database, and use the images to train the machine learning models. Given an image width *n* and an ECG recording, each annotated sample *x* has up to $\frac{n}{2}$ data points before and after *x* is captured, and we call this window *w*. If the number of data points in *w* is less than *n*, *w* is discarded and the next sample that has an annotation is analyzed. Next, create a blank (0 value) 1-channel image *I* of dimension (*n* × *h*), where *h* is the desired height of the image. Each column *c* represents a sample in *w*, and the row *r* in column *c* is the the approximate value of *w*_*c*_. For each columns *c* in image *I*, we fill a pixel white (value 255) at position *I*(*c, r*) where *r* defined as:


$$r\;=\;\left\lfloor w_{c}/d*h\right\rfloor$$

where *d* is the difference of the maximum limit and the minimum limit of the ECG, *w*_*c*_ is the *c*^th^ reading of *w*.

**Figure 2:**
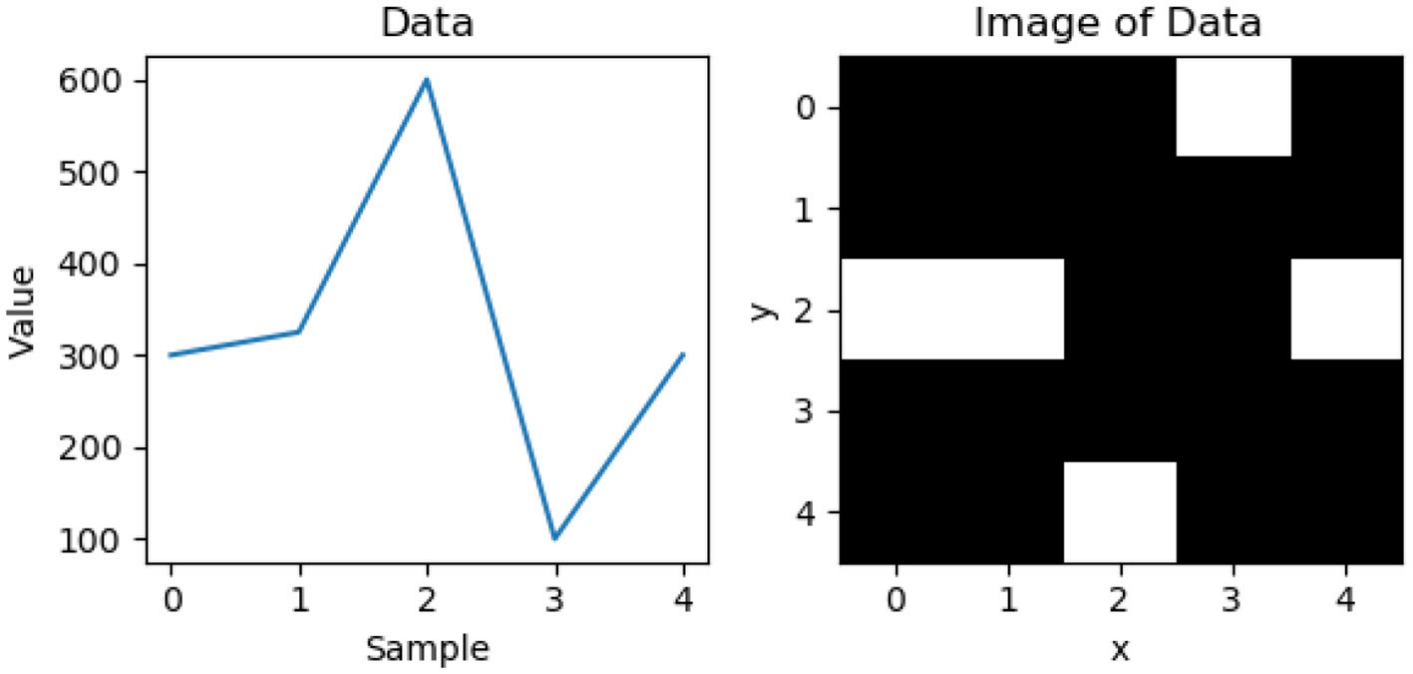
ECG to image conversion example.

As an example (Figure 2), consider the ECG sequence [300, 325, 600, 100, 300] with a minimum and maximum value of [0, 700] and an image height *h* = 5. The value *d* is calculated as 700 − 0 = 700. The first value reads 300 and *r* is calculated as $\left\lfloor \frac{300}{700}*5\right\rfloor =\left\lfloor 2.14\right\rfloor =2$. Repeat the same calculation for the rest of the sequence, and they are translated to the *y* values of the image as [2, 2, 4, 0, 2]. We then fill a pixel white at a position in the order it appears. That is, from the left most column we fill a pixel white at row 2, move one column to the right and fill a pixel at row 2, move one column right and fill row 4, and so on. The coordinate of pixels that needed to be filled for this example are (0, 2), (1, 2), (2, 4), (3, 0), (4, 2). The images are generated up-side down, but this is correct due to how image coordinate works. We can rework the algorithm so that it is human-readable, but this is unnecessary as the images are meant for the neural network. This method is an effective way to convert ECG signals into a 2-dimensional greyscale graph, while capturing the patterns in ECG. We chose this method because it allows the flexibility to adjust resolution of the 2D images, making it easier to meet the real-time challenges on edge devices.

Note that if the machine learning based arrhythmia detection is used in practice, signals that are generated by the ECG hardware may not be centered around the peak measurement, as shown in Figure 2. To address this issue, a random skew is applied to each signal during the data generation step: before the image is generated, the signal is randomly shifted $\pm \frac{n}{4}$ samples and the same image creation steps are applied as normal. Therefore, we generate two datasets, centered alignment (aligned) and skewed alignment (skewed). Both datasets are used to evaluate the proposed approach to provide a comprehensive evaluation.

### 4.2 Model architecture

The structure of this network is inspired by existing widely used CNN models, such as AlexNet (Krizhevsky et al., 2012) and VGGNet (Simonyan and Zisserman, 2014), with simplified architecture to enable execution on resource constrained devices and meet the real-time processing requirement. Most visual learning network uses a large amount of filters in the convolutional layers to learn features found in RGB images. Even with gray-scaled images, the number of filters can be moderate (about 32 filters) for these convolutional neural networks. Given that our image data are simple and have small dimensions, the goal is to build a CNN with simple structure to mitigate training overhead and meet the resource bounds on wearable devices. Specifically, we selected 3 sets of 2D convolutional and max pooling layers, with the number of filters of 8, 16, and 32, respectively. The first convolutional layer of 8 filters attempts to learn the pattern of the input image, and the subsequent convolutional layers will learn from those features. We also need to determine a suitable kernel size on the first convolutional layer so that the model can easily identify the ECG pattern. Initial experiments have been carried out to evaluate various kernel sizes, and we selected size of 6, 5, and 4 for the respective layers, which allows to detect the few features of the input image and done so in a timely manner. We selected these parameters for the network to additionally address the challenge of real-time processing. Table 2 shows the structure of the proposed CNN model.

**Table 2:** 2D CNN network architecture.

**Layer**	**Filters/nodes**	**Kernel size**	**Activation**
Input	(180, 64, 1)	-	-
Conv2D	8	(6,6)	ReLU
MaxPooling2D	-	-	-
Conv2D	16	(5,5)	ReLU
MaxPooling2D	-	-	-
Conv2D	32	(4,4)	ReLU
MaxPooling2D	-	-	-
Flatten	-	-	-
Dense	512	-	ReLU
Dense	256	-	ReLU
Dense	128	-	ReLU
Dense	15	-	SoftMax

To establish a baseline model to compare against, we used a simplified version of the CNN architecture presented in Jun et al. (2018). We keep the original architecture, but reduce the number of filters in each of the convolutional layers by $\frac{1}{8}$, in order to support resource-constraint environment. We use the same data processing method to prepare training/testing data for the network with the image dimension 128 × 128. The architecture of the baseline model is shown in Table 3.

**Table 3:** 2D CNN Baseline Network Architecture.

**Layer**	**Filters/nodes**	**Kernel size**	**Activation**
Input	(128, 128, 1)	-	-
Conv2D	8	(3,3)	ReLU
BatchNormalization	-	-	-
Conv2D	8	(3,3)	ReLU
BatchNormalization	-	-	-
MaxPooling2D	-	-	-
Conv2D	16	(3,3)	ReLU
BatchNormalization	-	-	-
Conv2D	16	(3,3)	ReLU
BatchNormalization	-	-	-
MaxPooling2D	-	-	-
Conv2D	32	(3,3)	ReLU
BatchNormalization	-	-	-
Conv2D	32	(3,3)	ReLU
BatchNormalization	-	-	-
MaxPooling2D	-	-	-
Flatten	-	-	-
Dense	512	-	ReLU
BatchNormalization	-	-	-
MaxPooling2D	-	-	-
Dense	15	-	SoftMax

### 4.3 Model evaluation

A comprehensive set of experiments were carried out on classifying ECG signals using the proposed model with both datasets with centered and skewed alignment, respectively. All experiments use a traditional training method of splitting the dataset into a 9:1 training/testing split. Both our proposed model and the baseline model were evaluated using the MIT-BIH dataset. The distribution of normal and arrhythmia samples in both the training and testing datasets is 66.6%:33.4%. All experiments were performed on a computer with an Intel i5 core @ 2.5 GHz, 8 GB RAM, and an NVIDIA Geforce 1050.

Table 4 shows the performance of the proposed CNN model on the testing dataset. Across a few different number of epochs, the network shows that it can provide good result within 10 epochs. On average, both networks that used aligned and skewed data points takes approximately 72 s to train per epoch and testing takes approximately 63 ms per batch of 512 samples. The testing time alone is fast enough for real-time classification, as it takes a 360 Hz ECG device about 2.7 ms to generate a single reading. In theory, a single sample would take 11 μ*s* under the same hardware that it was trained on, satisfying the requirement for real time classification. If places such as clinics and hospitals use similar hardware, then a deeper model is preferred because of its potentially higher accuracy.

**Table 4:** Performance of the proposed 2D model.

	**Aligned**			**Skewed**		
**Epochs**	**1**	**10**	**20**	**1**	**10**	**20**
Accuracy	0.956	0.968	**0.969**	0.905	**0.941**	0.939
Precision	0.962	0.969	**0.970**	0.921	**0.945**	0.943
Sensitivity	0.997	0.967	**0.969**	0.888	**0.937**	0.936
Specificity	0.994	0.997	**0.997**	0.994	**0.996**	0.995

Bold numbers shows the best performance in these epochs.

Table 5 shows the performance of the baseline model on the testing dataset. In comparison to the result of the proposed model in Table 4, the modified baseline model takes approximately 2–3 times long to complete its tasks, 161 second per epoch for training and 127 ms per batch of 512 samples for testing (about 24 μ*s* per item), but with similar results when compared to the proposed model. In both aligned and skewed ECG data, the differences in accuracy between the proposed and the baseline mode is about ±1%. These results indicate that the proposed model can perform the same task with less time.

**Table 5:** Performance of the Baseline 2D Model.

	**Aligned**			**Skewed**		
**Epochs**	**1**	**10**	**20**	**1**	**10**	**20**
Accuracy	**0.967**	0.966	0.939	0.894	0.935	**0.938**
Precision	**0.969**	0.966	0.940	0.903	0.937	**0.940**
Sensitivity	**0.964**	0.965	0.939	0.888	0.935	**0.937**
Specificity	**0.997**	0.997	0.995	0.993	0.995	**0.995**

Bold numbers shows the best performance in these epochs.

## 5 Workflow

To evaluate the proposed model's performance in a real-time setting, we have developed a computational workflow which can be used to test the feasibility of real-time processing. This workflow is divided into two components: the simulator and the detector.

This ECG simulator mimics the hardware that generates ECG readings from the ECG leads. It loads the entire prerecorded ECG into memory (about 9 MB in size for a MIT-BIH ECG record) and pushes a sample about every $\frac{1}{fs}$ second to a buffer *b* of size *n*, where *fs* is the recorded frequency. If the buffer *b* is full, it removes the oldest sample and pushes the new sample. During experiments, pushing a sample at the exact requested time of $\frac{1}{fs}$ seconds causes the whole workflow to run on average 10% longer than expected (i.e. an ECG recording of 30 seconds actually runs for 33 seconds). Because of this, the frequency for the simulator is adjusted by a factor of 0.9.

The detector component contains the neural network, which will detect ECG samples that it acquires from the simulator. First, it loads the pre-trained neural network and waits for the buffer *b* from the simulator to be full. Once the buffer *b* is full, the detector creates a copy of the buffer and we call this copy of the buffer *b*_*c*_. The detector may perform a check to determine if the signal *b*_*c*_ contains a beat at the center of the buffer. If the check determines that the signal is not centered, the signal is rejected and the detector process starts over. The classifier will generate an image *I* from the buffer *b*_*c*_ described in Section 4, then feed to the pre-trained network and output a result. This process continues until the simulator has no more samples. During the classification process, the detector will not check at the buffer *b* until its task is complete, and the detector will miss several samples that were pushed to the buffer *b* by the simulator as a result of this.

We can solve the issue of missing samples during classification by creating a buffer of buffers (*b*_*b*_). However, this is unnecessary. If this buffer of buffers creates copies of the buffer *b* at every sample that is generated by the simulator, the buffer *b*_*b*_ will eventually contain the whole recording and will cause delay between when the sample is copied and when the same sample is classified. We can also create a delay between samples being pushed into this buffer. But if this timing is too short, it can create the problem of eventually containing the whole recording. Conversely, if the timing is too long such that the detector already finishes its task, the detector can get data from the buffer *b* directly.

The experiments for the workflow were carried out on a laptop with a i7–8565U CPU @ 1.8 GHz, 8 GB of RAM, and Ubuntu 20. This was done so that timing for the detection component is similar to machines that are found in a hospital environment. This minimum sleep time causes the the whole workflow to stall (in particular the simulator thread) until the sleep function exits and throw off the timing results.

**Figure 3:**
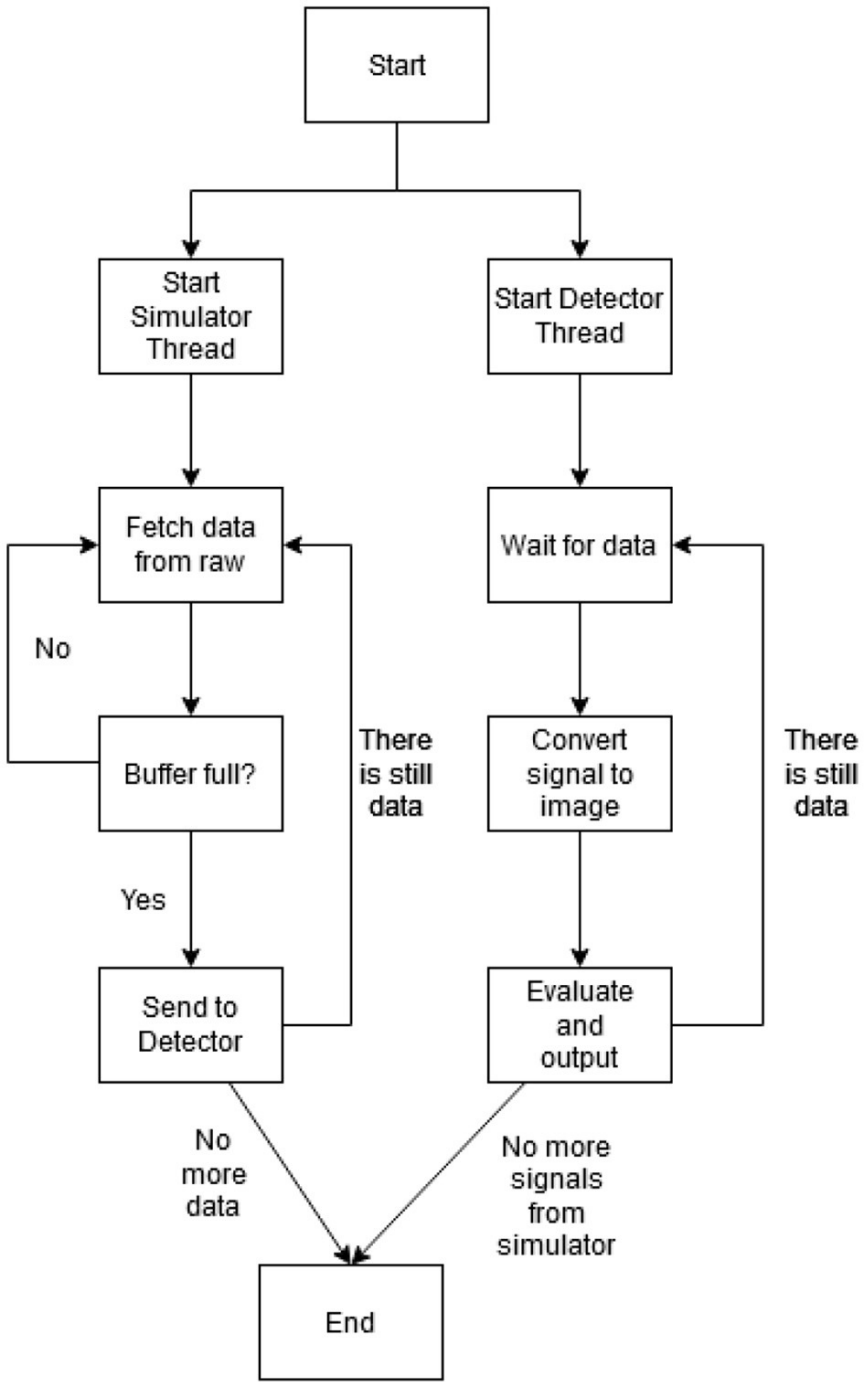
The ECG detection workflow diagram.

Table 6 shows the runtime result of the first 30 s of a random MIT-BIH record in the testing dataset using the workflow shown in Figure 3 averaged across 10 runs. The records can be picked randomly, as all of the records in the MIT-BIH arrhythmia database are at the same frequency. We only need to look at the run time of the implemented features of this workflow. “Check Center” is a simple check that determines if the input signal contains a heartbeat that is located near the center. This check is optional and can be performed to lessen the amount of outputs.

**Table 6:** Classification (C) average timing.

**Model**	**Check center**	**Average classification time (s)**	**Average number of samples removed during classification**
Baseline	Yes	0.024024	8.648583
	No	0.024731	8.903034
Proposed	Yes	0.020661	7.438066
	No	0.020651	7.434187

Overall, both the proposed and baseline model shows great result in terms of the computational performance. Table 6 shows the classification time for a single image, and this shows that a small, compact model (i.e., the proposed model) slightly outperforms a larger model (baseline). Other processes such as image conversion (Table 7) and check center (Table 8) can be completed within one sample the ECG recording frequency, therefore, they do not cause additional overhead for the integrated system.

**Table 7:** Image conversion average timing.

**Model**	**Check center**	**Average time for image conversion (s)**	**Average number of samples removed during image conversion**
Baseline	Yes	0.00013	0.046923
	No	0.000148	0.053171
Proposed	Yes	0.000177	0.06341
	No	0.000182	0.06538

**Table 8:** Check center average timing.

**Model**	**Check center**	**Average time for check center (s)**	**Average number of samples removed during check center**
Baseline	Yes	0.001973	0.710142
	No	-	-
Proposed	Yes	0.002218	0.798551
	No	-	-

## 6 Integrated system evaluation

Using the open-sourced MIT-BIH Arrhythmia Database (Goldberger et al., 2000), we have demonstrated the effectiveness of the proposed CNN model in terms of detecting signals with arrhythmia. The next step is to demonstrate the applicability of this method in real-world scenario.

**Figure 4:**
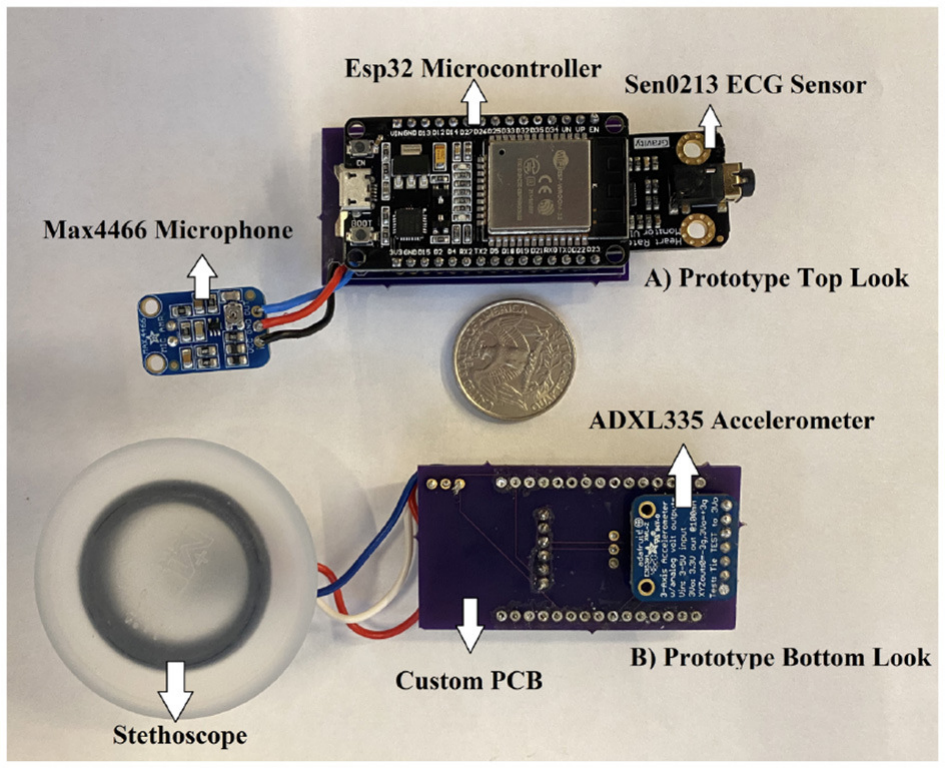
Prototype of wearable heart monitoring device.

We have previously developed a portable device (Yakut et al., 2022) that captures the sound of the heart beat (Phonocardiogram, PCG) and the chest vibration generated by the heart (Seismocardiography, SCG), along with the heart's electrical activity (Electrocardiogram, ECG). The electrodes are intended to be placed on three different location of the patient's chest, two on the left and one on the right, along with a microphone for the PCG data collection. The hardware also include a Bluetooth module that can communicate to a mobile device or PC, and a back-up storage via a micro-SD card. The prototype is shown in Figure 4.

Using the 360 Hz recording we have collected in the lab setting (Yakut et al., 2022), the proposed model from Section 4 and the workflow mentioned in Section 5, the model returns good results with an average confidence of 97% (weighted confidence 97%±0.02%). Note that the confidence score from the classification is an indication of how likely (probability) the predictions of a machine learning algorithm are correct. The classification mostly returns *N*, indicating that the model, without having to train in-between beats, can correctly identify a beat. Given that the data was collected from a healthy individual, all testing data were normal signals. A sample result with the first 10 seconds of the newly recorded signals is shown in Table 9. The table shows the ID of the heartbeat peaks, the position of the heartbeat peak in the whole signal (360Hz, 10 seconds), classification results, as well as the confidence scores. Note that these results are denoised with discrete wavelet transform and contain a center check to help align the signals.

**Table 9:** Small sample of results for 360 hz recording with center check.

**ID**	**Location**	**Classification**	**Confidence**
0	249–429	N	99.99%
1	458–638	N	100.00%
2	465–645	N	100.00%
3	472–652	N	99.99%
4	685–865	N	99.99%
5	692–872	N	99.33%
6	699–879	N	99.60%
7	919–1099	N	99.99%
8	927–1,107	N	99.99%
9	934–1,114	N	54.41%
10	1,163–1,343	N	99.99%
11	1,170–1,350	N	100.00%
12	1,177–1,357	N	99.88%
13	1,440–1,620	N	100.00%
14	1,448–1,628	N	99.96%
15	1,455–1,635	N	97.18%
16	2,036–2,216	N	100.00%
17	2,043–2,223	N	99.80%
18	2,335–2,515	N	99.99%
19	2,640–2,820	N	100.00%
20	2,648–2,828	N	99.99%

**Figure 5:**
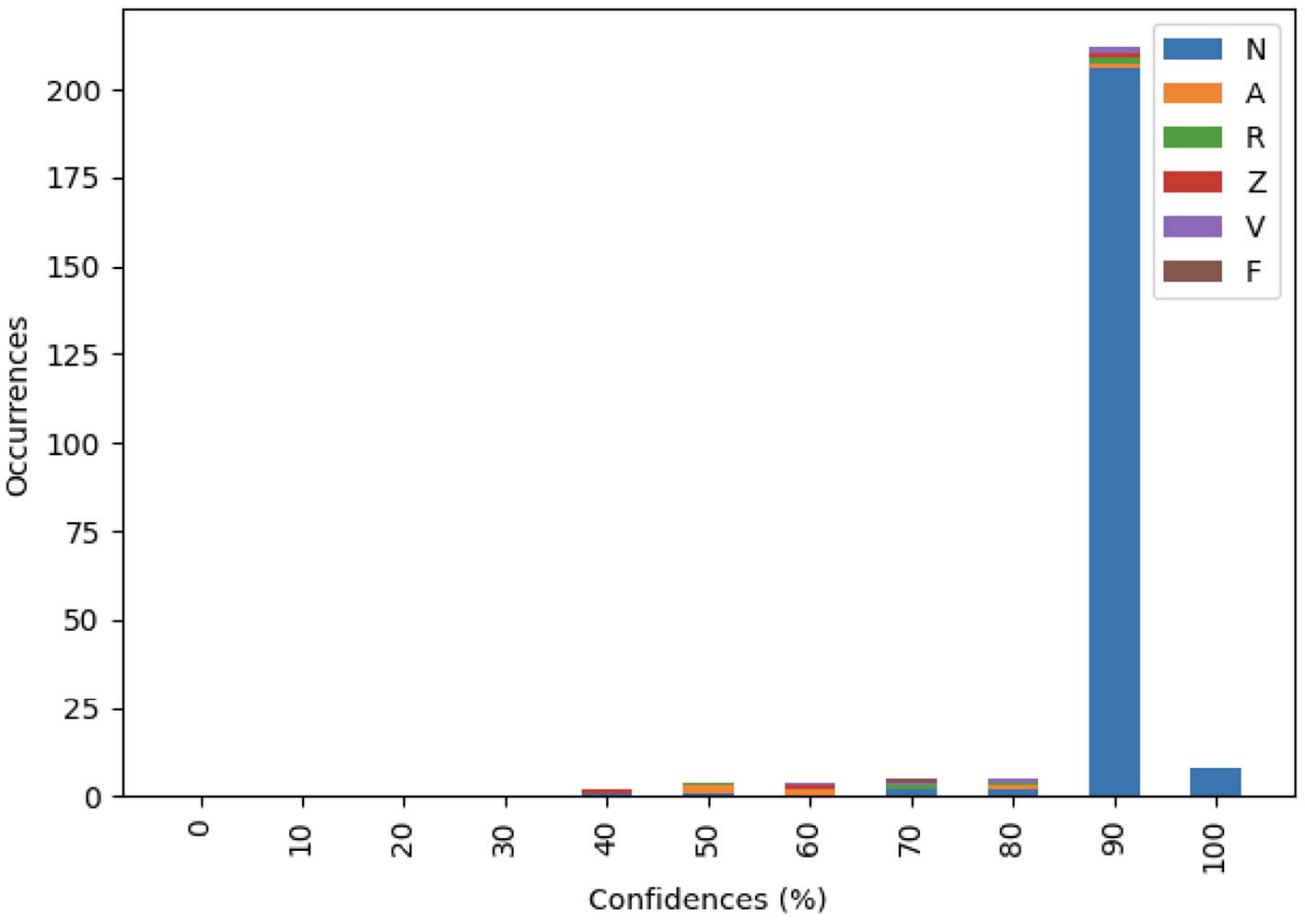
Distribution of the 360 Hz recording by confidence.

We also performed the same experiment mentioned above on the whole recording. As shown in Figure 5 and Table 10, the proposed model shows high confidence in detecting normal heart beat.

**Table 10:** Distribution of the 360 Hz recording.

**Classification**	**Occurrences**
A	6
F	1
N	220
R	5
V	5
Z	3

In term of performance, the workflow runs within the expected time frame. The whole workflow takes 64 s to complete, it detected and classified 240 samples, most of which are detected almost immediately after the detector finished its previous task. On average, the workflow skips only 7.4 samples (about 0.02 s) per classification, demonstrating the potentials for applying in a real-time environment.

## 7 Conclusion

Arrhythmia such as atrial fibrillation is a common cause of death in the United States. While the most common way to diagnose arrhythmia is through an ECG reading, a challenge that comes from this method of diagnosis is that it requires a trained medical profession to evaluate the ECG reading. The development of a machine assisted method can speed up the diagnostic process and potentially reduces fatality. In this paper, we present a convolutional neural network based method that classifies a short ECG sample. Additionally, we have created a workflow that tests the model's potential in a real-world environment. Our result shows that a 2D convolutional neural network that uses image representation of the input signal shows a high degree of confidence of around 90%. The proposed workflow determines that the proposed network is capable of classifying ECG samples, and its performance is feasible to implement real-time arrhythmia detection on wearable devices.

For future work, we will build on top of the classification results, and develop feature extraction methods and machine learning models for anomaly detection. Classification provides a good guidance on diagnosis, because it classifies data samples to a number of known conditions. However, in a real-time setting, it might not be necessary to diagnose the underlying problems. Instead, being able to quickly detect abnormal heart rhythms is more critical, i.e., anomaly detection. Therefore, a binary classifier which detects anomaly signals from the normal ones is the essential part of real-time monitoring and detection of arrhythmia. In addition, we will develop real-time sensing techniques to collect data at real-time, and evaluate our models using data collected from patients.

## Data availability statement

The raw data supporting the conclusions of this article will be made available by the authors, without undue reservation.

## Author contributions

TV: Data curation, Formal analysis, Methodology, Software, Validation, Visualization, Writing—original draft. TP: Data curation, Methodology, Writing—review & editing. KY: Data curation, Writing—review & editing. MU: Data curation, Writing—review & editing. WX: Conceptualization, Writing—review & editing. FH: Conceptualization, Writing—review & editing. RH: Conceptualization, Writing—review & editing. XZ: Conceptualization, Formal analysis, Methodology, Supervision, Writing—original draft, Writing—review & editing.
